# Suction mini-percutaneous nephrolithotomy versus standard percutaneous nephrolithotomy for the management of 2–4 cm kidney stones: study protocol for an international, multicenter, parallel-group, noninferiority, randomized controlled trial

**DOI:** 10.1007/s00345-026-06302-7

**Published:** 2026-02-26

**Authors:** Wen Zhong, Kehua Jiang, Xuepei Zhang, Chi Ho Leung, Wei Zhu, Zhanping Xu, Guofu Pang, Zhongyi Sun, Fan Cheng, Jin Li, Jie Chen, Yuanwei Li, Houmeng Yang, Shulian Chen, Chuanxun Wu, Rui Jia, Jin Zhu, Jorge Gutierrez-Aceves, Emanuele Montanari, Shabir Almousawi, Iliya Saltirov, Marcin Popiolek, Albert Aquino, Giorgio Mazzon, Simon Choong, Haibo Xi, Steffi Kar Kei Yuen, Guohua Zeng

**Affiliations:** 1 Department of Urology, The First Affiliated Hospital of Guangzhou Medical University, Guangzhou, China; 2 Department of Urology, Shufu People’s Hospital, Kashgar Prefecture, Xinjiang Uygur Autonomous Region China; 3 Department of Urology, Guizhou Provincial People’s Hospital, Guiyang, China; 4 Department of Urology, The First Affiliated Hospital of Zhengzhou University, Zhengzhou, China; 5 SH Ho Urology Centre, Department of Surgery, The Chinese University of Hong Kong, Hong Kong, China; 6 Department of Urology, Foshan Hospital of Traditional Chinese Medicine, The Eighth Clinical Medical College of Guangzhou University of Chinese Medicine, Foshan, China; 7 Department of Urology, Zhuhai People’s Hospital, Zhuhai Hospital Affiliated of Jinan University, Zhuhai, China; 8 Department of Urology and Carson International Cancer Center, Shenzhen University General Hospital, Shenzhen, China; 9 Department of Urology, Renmin Hospital of Wuhan University, Wuhan, Hubei China; 10 Department of Urology, Central Hospital of Xiangtan, Changsha, China; 11 Department of Urology, Jiangxi Provincial People’s Hospital, The First Affiliated Hospital of Nanchang Medical College, Nanchang, China; 12 Department of Urology, Hunan Provincial People’s Hospital, Changsha, China; 13 Department of Urology, Huamei Hospital, University of Chinese Academy of Sciences, Ningbo, China; 14 Department of Urology, Affiliated Hospital of Zunyi Medical University, Zunyi, China; 15 Department of Urology, The First People’s Hospital of Fuquan City, Fuquan, China; 16 Department of Urology, Qiannan Buyi and Miao Autonomous Prefecture Hospital, Duyun, Guizhou Province China; 17 Department of Urology, the Second Affiliated Hospital of Soochow University, Suzhou, China; 18 Department of Urology, Cleveland Clinic, Cleveland, Ohio USA; 19 Department of Urology, University of Milan - Fondazione Ca’Granda IRCCS Ospedale Maggiore Policlinico Milano, Milan, Italy; 20 Department of Urology, Sabah Al-Ahmad Urology Centre, Kuwait City, Kuwait; 21 Department of Urology and Nephrology, Military Medical Academy, Sofia, Bulgaria; 22 Department of Urology, Örebro University Hospital, Örebro, Sweden; 23 Department of Urology, Jose R. Reyes Memorial Medical Center, Manila, Philippines; 24 Department of Urology, University College London Hospitals, London, UK; 25 Department of Urology, The First Affiliated Hospital of Nanchang University, Nanchang, China; 26 European Association of Urology Section of Endourology (ESEUT), Arnhem, The Netherlands

**Keywords:** Suction, Percutaneous nephrolithotomy, Mini PCNL, Renal stones

## Abstract

**Purpose:**

Percutaneous nephrolithotomy (PCNL) is recommended as the first-line treatment choice for the management of kidney stones larger than 2 cm. However, tract-related complications have always been a concern, especially in standard PCNL where a larger tract > 24Fr is established. The mini-PCNL technique with smaller tract (≤ 18 Fr) has been introduced to decrease tract related complications. Yet, with a smaller mini-PCNL tract, fragments must be fragmented into smaller pieces before extraction, potentially prolonging the operative time. Moreover, if the outflow of irrigation fluid is insufficient via the smaller tract, the intrarenal pressure (IRP) can build up, leading to pyelovenous backflow and infectious complications – an issue theoretically mitigated by the addition of suction techniques. However, high-level evidence comparing suction mini-PCNL outcomes to standard PCNL is still lacking.

**Methods:**

The present multicenter, international, randomized controlled noninferiority trial compares suction mini-PCNL to standard PCNL in the management of 2–4 cm kidney stones. The primary outcomes are the immediate postoperative stone-free rate (SFR) and operative time, while secondary outcomes include the final SFR at 1 month, postoperative pain score, length of hospital stay, postoperative complications, and quality of life.

**Results:**

A total of 960 patients from 20 urological centers will be randomized to receive either suction mini-PCNL or standard PCNL. Intention-to-treat analysis will be performed. For SFR, non-inferiority will be concluded if the lower bound of the 95% confidence interval for the absolute risk difference exceeds − 10%. For operative time, non-inferiority will be concluded if the lower bound of the 95% confidence interval for the mean difference exceeds − 10 min. For all other analyses, a two-sided p-value of < 0.05 will be regarded as statistically significant. Subgroup analyses will be performed to explore potential effect modification by stone location and stone density.

**Conclusion:**

The aim of this trial is to generate high-level evidence regarding the noninferiority of suction mini-PCNL compared to standard PCNL for 2–4 cm renal stones. The trial is registered on ClinicalTrials.gov as NCT05088213.

## Introduction

Percutaneous nephrolithotomy (PCNL) is recommended as the first-line treatment choice for kidney stones larger than 2 cm in international guidelines [1–3]. However, tract-related complications affecting renal parenchymal and blood vessels have always been a concern [4–6]. The mini-PCNL technique which uses a smaller tract (≤ 18 Fr) has been introduced to decrease tract-related complications [7–9]. Nevertheless, the smaller mini-PCNL tract necessitates fragmentation of stones into smaller pieces before extraction, potentially prolonging the operative time. Additionally, higher pressure irrigation is often required to flush out fragments by vacuum cleaner effect [10]. If the drainage of irrigation fluid is insufficient via a smaller tract, the intrarenal pressure (IRP) may build up, leading to pyelovenous backflow and infectious complications [11].

Suction application in mini-PCNL has the potential to enhance stone fragments removal efficiency, maintain low IRP, and decrease potential postoperative infection complications [12–14]. In the pre-suction era, mini-PCNL achieved noninferior stone-free rates (SFR) and operative times compared to standard PCNL, along with benefits of reduced bleeding, less postoperative pain, and shorter hospital stay [15]. However, high-level evidence regarding whether suction mini-PCNL can attain comparable outcomes to standard PCNL is still lacking.

Therefore, we have designed this multicenter, international, randomized controlled noninferiority trial comparing suction mini-PCNL with standard PCNL in the management of 2–4 cm kidney stones.

## Study objectives

The objective of the present study is to determine whether suction mini-PCNL is noninferior to standard PCNL in terms of surgical efficiency in the management of 2–4 cm kidney stones.

The primary outcomes are the immediate postoperative stone-free rate (SFR) and operative time. Secondary outcomes include the final SFR at 1 month, length of hospital stay, postoperative complications, and quality of life.

## Study design and methods

### Study overview

The present study is an international, multicenter, prospective, parallel group, noninferiority, randomized controlled trial.

### Setting

This study will be conducted in accordance with the principles of the Declaration of Helsinki. Approval was granted by the Medical Research Ethics Committee of The First Affiliated Hospital of Guangzhou Medical University: #2021(81). The study will be carried out in 20 urological institutes across the world, including 6 in Europe, 4 in America and 10 in Asia. Each participating urologist is required to possess significant experience of both suction mini-PCNL and standard PCNL, conducting ≥ 100 cases per annum.

### Sample size

The reported SFRs following suction mini-PCNL and standard PCNL for 2–4 cm renal stones are 83% and 86% respectively [3, 10, 13, 15]. We hypothesize that the SFR following suction mini-PCNL is noninferior to standard PCNL. The noninferiority margin is prespecified at -10%, based on clinical consensus and prior randomized controlled trial [15]. Assuming a one-sided α of 0.025, a power of 80%, and a noninferiority margin of -10% [15], the minimum required sample size per group is calculated to be 419. Considering an anticipated withdrawal and loss to follow-up rates of approximately 10%, this is adjusted to 480 participants per group, resulting in a total sample size of 960.

The reported operative times for mini-PCNL and standard PCNL in the management of 2–4 cm kidney stones are comparable [15]. Since suction mini-PCNL is faster than mini-PCNL [13], we conservatively assume that there is no difference in operative time between suction mini-PCNL and standard PCNL. The reported standard deviation is approximately 15 min [13, 16]. Our hypothesis is that the operative time for suction mini-PCNL is noninferior to that of standard PCNL. Assuming a one-sided α of 0.025, a power of 80%, and a noninferiority margin of -10 min, the minimum required sample size per group is calculated to be 43, which is smaller than the sample size determined based on SFR.

Thus, the final total sample size is set at 960.

### Patients population

The inclusion criteria are as follow: (1) Adults aged 18–70 years; (2) American Society of Anesthesiology (ASA) score of 1–2; (3) Kidney stones measuring 2–4 cm in diameter on non-contrast computer tomography; (4) Ability to provide written informed consent and adhere to trial requirements.

The exclusion criteria are as follow: (1) Anatomy abnormalities, such as urinary diversion, horseshoe kidney, transplanted kidney, or ureteropelvic junction obstruction; (2) Untreated acute urinary tract infection (UTI); (3) Patients on anticoagulant/antithrombotic therapy, or with uncorrected coagulopathies; (4) Patients who decline trial enrolment or cannot adhere to trial requirements.

### Screening and enrollment of participants

All participating centers will follow a standardized process to conduct this study. Clinicians or designated personnel will assess patients with suspected renal stones as part of routine clinical practice. Once renal stones are confirmed by CT scan and patients are fully evaluated for PCNL, eligible patients screened from the inclusion and exclusion criteria will be well informed of the trial and offered an information leaflet, detailing the potential benefits and drawbacks of the two different PCNL techniques. Patients will have ample opportunity to discuss with the local clinical team, and those who choose to participate will be able to do so. A log will be kept of all patients assessed to document reasons for their non-inclusion in the study to inform the CONSORT diagram. In addition to regular coverage, additional medical insurance and perioperative care would be provided for these candidates. Signed informed consent forms will be obtained from all participants prior to any study-specific procedures. Following consent, participants will be randomized to one of the two treatment groups.

### Randomization and allocation and blinding

Participants will be randomized in a 1:1 ratio to either standard PCNL or suction mini-PCNL. Stratified randomization will be performed according to the participating centers. Randomization sequence generation will be performed electronically using random block sizes of 4 and 6 prior to patient enrolment. Random sequence allocation and concealment will be implemented using consecutively numbered, opaque, tamper-proof envelopes.

Patients will be blinded to the intervention, however blinding of surgeons will not feasible due to the nature of the procedures. Radiologists evaluating the postoperative CT scans will be blinded to the intervention received. The postoperative clinical assessment will be performed by investigators blinded to the intervention and not involved in the surgical interventions. Blinded statistical analyses will be conducted with the code of intervention arm assignment remaining concealed until the analyses and interpretation have been completed.

### Surgical protocol

A standardized operative protocol will be established to ensure uniformity across all centers. Periodic protocol monitoring visits will be conducted to ensure its adherence at all participating centers.

#### Preoperative evaluation and preparation

In addition to the routine blood and urine examination, a non-contrast CT (NCCT) scan with 2 mm slice thickness is required for all patients. Stone characteristics, such as burden (largest diameter) and density (Hounsfield Unit, HU), will be measured from NCCT.

A single prophylactic antibiotic dose (200 mg cefuroxime or 500 mg levofloxacin, or according to local antibiograms at the discretion of the operating surgeon) will be administered to all patients 0.5 h before surgery. Patients with positive urine culture will receive culture-sensitive antibiotics for 5–7 days prior to procedure.

All procedures will be performed under general anesthesia. A 5Fr open-ended ureteral catheter will be inserted into the target ureter in lithotomy position, followed by a 16Fr Foley catheterization for bladder drainage. The patient will then be turned prone for PCNL access tract creation and lithotripsy.

#### PCNL procedures

Ultrasound-guided puncture to the target calyx will be performed using an 18-gauge coaxial needle. Retrograde pyelogram via the ureteral catheter will confirm the puncture position, followed by guidewire insertion. Tract dilatation over guidewire will be accomplished using fascial dilators.

For standard PCNL, 24Fr sheath will be inserted, and 20Fr nephroscope (Richard Wolf, Germany) will be used. Renal stones will be fragmented and aspirated using an ultrasonic-lithotripter (LithoClast Master, EMS or ShockPluse-SE, Olympus).

For suction mini-PCNL, 18Fr suction sheath (Wellead, China) will be inserted, and 12Fr mini-nephroscope will be used. Renal stones will be fragmented by pneumatic lithotriptor or Ho: YAG laser (1.0–2.0 J ×20–30 Hz), and stone fragments will be aspirated out via the suction sheath.

Upon conclusion of the procedure, fluoroscopy will be utilized to detect any residual stones. If present, further attempts, including the possible creation of additional tracts, will be made to remove all residual fragments. Double-J stent and/or nephrostomy tube will be inserted at the discretion of the operating surgeon. Tubeless PCNL may be feasible if there is no significant residual stone, bleeding, collecting system perforation, or ureteral obstruction.

### Postoperative evaluation and follow-up

Operative details including operative time will be timely documented. All intraoperative complications including bleeding, collecting system perforation, organ injury or other perioperative complications will be evaluated using the Clavien-Dindo grading system.

Complete blood count and electrolyte biochemical tests will be performed within 2 h after surgery. Patients with a hemoglobin level < 70 g/l will receive blood transfusion.

KUB will be obtained on the morning of postoperative day one to evaluate the stone-free status, stent and nephrostomy tube status. A low-dose NCCT scan with 2 mm slice thickness will be conducted within 72 h after the operation to evaluate the postoperative immediate SFR. If residual fragments ≧4 mm are present, any information concerning auxiliary treatments will be recorded, including the size of residual stones, type of auxiliary treatment and final outcome.

If nephrostomy tube is placed, it will be removed in the absence of significant bleeding or complications, followed by patient discharge. The double-J stent will be removed after 2 weeks.

Pain visual analog scale (VAS) will be evaluated at three time points, namely pre-operatively, post-operatively 6 h, and follow up at 1 month respectively. Postoperative analgesia requirement will be documented to reflect postoperative pain and recovery. Quality of life (QoL) scores will be assessed by Wisconsin Stone Quality of Life questionnaire (WISQOL) pre-operatively, as well as 4 weeks postoperatively [17].

### Data collection and statistical analysis

#### Data collection and quality control

Patients’ demographics and all clinical outcomes will be recorded in the pre-established case report forms (CRF). Two urologists will take turns recording and verifying work for each recruited case. The data of each participating center will be uploaded and summarized weekly, and on-site verification will be conducted periodically to ensure data authenticity and accuracy. Reassessment will be required if summarized data deviate significantly from the median level in the preliminary statistics analysis.

Stone-free status will be evaluated by blinded radiologist from 2 mm slice NCCT scan performed postoperatively within 72 h and at 1 month. Stone size will be defined as the largest diameter for a single stone or the sum of the largest diameters for multiple stones. Stone size and Hounsfield units (HU) will be measured consistently using the same software across all centers. S.T.O.N.E. score will be used for evaluation of stone complexity [18]. Operative time will be calculated from the percutaneous puncture to completion of double-J or nephrostomy tube placement. Hospital stay will be calculated from the day of surgery to the day of discharge and rounded up to the nearest whole day. Intraoperative and postoperative complications will be documented and graded according to the Clavien-Dindo classification.

Each recruited case will have three CRFs completed by its participating center: one at randomization, one after the intervention, and one at 1-month follow-up, including details of any additional interventions and complications. Table 1 outlines the schedule for outcome assessment and data collection (Fig. 1).

**Table 1:** Schedule for data collection and outcome assessment

Outcome measure	Timing				
	Recruitment	Intervention			Follow-up
		Intraoperative	Postoperative	Upon discharge	1 month
Baseline assessment					
Age	√				
Stone size	√				
ASA score	√				
Laboratory tests	√		√		√
Stone evaluation					
KUB X-Ray	√		√		
NCCT	√		√		√
Quality of life score	√				√
Pain score	√		√		√
Operative characteristics					
Operation time		√			
Puncture guidance		√			
Puncture site		√			
Tract size		√			
Tract number		√			
Tube status		√			
Hospital stay				√	
Complications					
Bleeding		√	√		
Infection		√	√		
Organ injury		√	√		
Others		√	√		√
Immediate stone-free status			√		
Final stone-free status					√

*ASA * American Society of Anesthesiologists, *KUB* kidney ureter bladder, *NCCT* non contrast computer tomography

#### Study flow chart

**Fig. 1:**
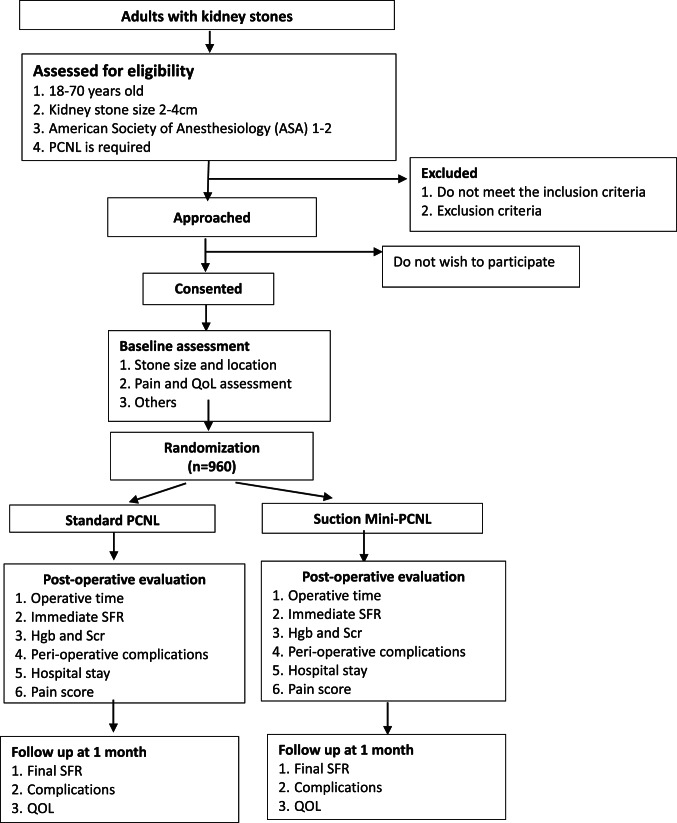
Study flow chart. *PCNL* percutaneous nephrolithotomy, *QOL* quality of life, *SFR* stone-free rate, *Hgb* hemoglobin, *Scr* serum creatinine

#### Outcome measures

##### Primary outcomes

The co-primary outcomes are the immediate postoperative stone-free rate (SFR) and operative time. Immediate postoperative SFR is defined as the absence of residual stone or fragments < 2 mm on low-dose NCCT scan within 72 h after operation. Operative time is defined as the time from percutaneous puncture to completion of double-J or nephrostomy tube placement.

Secondary outcomes are as follows:

- Final SFR: defined as no residual stone or fragments <2 mm on NCCT (2 mm slice) at 1 month post intervention.
- Pain VAS score.
- Length of hospital stay: calculated from the day of surgery to the day of discharge, rounded to the nearest whole day.
- Postoperative complications up to 1 month post-intervention, evaluated using the Clavien-Dindo classification.
- QoL will be assessed preoperatively and at 1 month postoperatively by the WisQoL questionnaire.

#### Statistical analysis

Intention-to-treat analysis will be performed. All enrolled participants with complete data will be included in the analysis. Statistical analyses will be conducted using Statistical Package for Social Sciences (SPSS) 22.0 or above and R software version 4.3.1 or above (R Foundation for Statistical Computing). Continuous data will be presented as median (IQR) and analyzed using Student’s t test or Mann-Whitney U test, as appropriate. Categorical data will be expressed as n (%) and compared using the χ2 test or Fisher’s exact test, as appropriate.

Pre-specified sensitivity analyses will be performed to assess the robustness of the primary findings under alternative, clinically plausible noninferiority margins. These sensitivity analyses will include SFR noninferiority margins of − 5% and operative time noninferiority margins of − 5 and − 10 min, respectively. These analyses will be exploratory in nature and are intended to evaluate the consistency of conclusions across different noninferiority thresholds, rather than to redefine the primary hypotheses or decision criteria. Subgroup analyses will be performed to explore potential effect modification by stone location, stone density and lithotripsy energy modality. Logistic regression and linear regression models including a treatment-by-subgroup interaction term will be used to evaluate the interaction effect on SFR and operative time, respectively.

This trial includes two co-primary endpoints: postoperative SFR and operative time. To control the overall type I error rate, a joint noninferiority testing strategy based on the intersection–union principle will be applied. Noninferiority of suction mini-PCNL will be concluded only if both co-primary endpoints independently satisfy their respective noninferiority criteria. Specifically, for SFR, non-inferiority is defined if the lower bound of the 95% confidence interval for the absolute risk difference exceeds − 10%. For operative time, non-inferiority is defined if the lower bound of the 95% confidence interval for the mean difference exceeds − 10 min. For all other analyses, a two-sided p-value of < 0.05 will be regarded as statistically significant.

## Discussion

Endourology has witnessed monumental technological advances, particularly with the introduction of suction in lithotripsy interventions and miniaturization of surgical equipment in the recent years.

Several studies have compared the clinical outcomes of mini-PCNL with those of standard PCNL, but reported inconsistent findings. The general consensus is that mini-PCNL holds the advantage of less invasiveness over standard PCNL, accounting for less blood loss and shorter hospital stay. However, the postoperative SFR of mini-PCNL is similar to standard PCNL for moderate-sized kidney stones [3, 10, 15]. The relatively longer operative time in mini-PCNL remains a topic of concern [10]. Since complication rates generally increase with prolonged operative time while SFR decreases, improving lithotripsy efficiency in mini-PCNL is an urgent priority.

The suction-assisted technique in lithotripsy has gained popularity in both PCNL and retrograde intrarenal surgery (RIRS) [19, 20]. Suction technique in mini-PCNL enables active aspiration of stone fragments, dust, blood clots, thereby maintaining excellent visualization at a low intrarenal pressure level, preventing stone migration, and facilitating the stone removal process, while mitigating the limitations of conventional mini-PCNL [13].

Beyond enhancing stone removal efficiency in mini-PCNL, suction can improve SFR, shorten operative time and reduce the need for auxiliary procedures [21, 22].

Concerning postoperative complications, existing literature has demonstrated that the overall complication rate in the suction mini-PCNL group is significantly lower than that of the conventional mini-PCNL group, particularly for postoperative fever, septic shock and blood loss [21, 22]. The well controlled IRP and shorter operative time by suction may account for the reduction in complication rates compared to conventional mini-PCNL [12].

To date, there is no large sample size randomized controlled trial (RCT) comparing suction mini-PCNL with standard PCNL. Given the promising outcomes of suction application in mini-PCNL, our study aims to compare suction mini-PCNL with standard PCNL for the treatment of 2–4 cm renal stones. The co-primary outcomes will assess the noninferiority of suction mini-PCNL versus standard PCNL in terms of SFR and operative time.

Complication profiles are also critical for evaluating PCNL; thus, secondary outcomes include postoperative complications, postoperative pain, length of hospital stay and QoL, on top of final SFR at 1 month. Despite the robust trial design, there are potential limitations, such as possible heterogeneity in terms of patient selection and treatment protocols (e.g. different lasers, energy modalities utilized, surgeon’s experience).

As an academic organization dedicated to urolithiasis prevention and treatment, the International Alliance of Urolithiasis (IAU) is committed to conducting this well-designed RCT to generate robust evidence to guide clinical practice. If suction mini-PCNL is proven non-inferior to standard PCNL in terms of SFR and operative time, this study will establish suction mini-PCNL as a feasible treatment option to standard PCNL with comparable efficacy and safety, particularly in patients at higher bleeding risks or seeking rapid recovery.

To the best of our knowledge, the present study is the largest RCT to compare the efficacy and safety of suction mini-PCNL with standard PCNL for 2–4 cm kidney stones. Our findings will advance renal stone treatment, optimize clinical strategies, and enhance the healthcare services quality, ultimately benefiting patients.

## Data Availability

The data that support the findings of this study are available from the corresponding author upon reasonable request.
